# Considering Exposure Assessment in Epidemiological Studies of Chronic Health in Military Populations

**DOI:** 10.3389/fpubh.2020.577601

**Published:** 2020-10-06

**Authors:** Amy L. Hall, Mary Beth MacLean, Linda VanTil, David Iain McBride, Deborah C. Glass

**Affiliations:** ^1^Research Directorate, Veterans Affairs Canada, Charlottetown, PE, Canada; ^2^School of Rehabilitation Therapy, Queen's University, Kingston, ON, Canada; ^3^Department of Preventive & Social Medicine, University of Otago, Dunedin, New Zealand; ^4^Public Health and Preventive Medicine, Monash University, Melbourne, VIC, Australia

**Keywords:** exposure assessment, military populations, veteran health, military health, military epidemiology

## Abstract

Exposure assessment is an important factor in all epidemiological research seeking to identify, evaluate, and control health risks. In the military and veteran context, population health research to explore exposure-response links is complicated by the wide variety of environments and hazards encountered during active service, long latency periods, and a lack of information on exposures in potentially vulnerable subgroups. This paper summarizes some key considerations for exposure assessment in long-term health studies of military populations, including the identification of hazards related to military service, characterization of potentially exposed groups, exposure data collection, and assignment of exposures to estimate health risks. Opportunities and future directions for exposure assessment in this field are also discussed.

## Introduction

In occupational studies, a lack of exposure data is often cited as the primary limitation in establishing exposure-response relationships between environmental hazards and subsequent health effects. Military-focused occupational health-related research is additionally complicated by the wide variety of environments and hazards encountered during active service. Military members across forces globally may encounter hazardous exposures during deployment (e.g., smoke, prophylactic pesticides), combat (e.g., chemical warfare agents, depleted uranium), basic training and job tasks (e.g., diesel engine exhaust, extreme physical loads), and environmental circumstances (e.g., dusts, infectious agents, temperature, and noise extremes) (1–4).

Military recruits are likely to be healthier than the general population from which they are recruited, recognized in epidemiological studies as the “healthy worker” (5) or “healthy soldier” (6) effect. The “healthy warrior effect,” where deployed defense force members tend to be healthier than those who are not deployed, has also been noted (7). However, research has also demonstrated that a range of chronic health conditions are more commonly reported in veterans compared to their general population counterparts, such as musculoskeletal issues, cancer, hearing problems, gastrointestinal problems, and mental health conditions (8–13).

Exposure assessment is an important factor in all epidemiological research seeking to identify, evaluate, and control health risks. It is particularly important in the study of chronic health outcomes, where latency periods can be long and exposures must be assessed over an extended period of time, often retrospectively. Strong exposure assessment requires both data to characterize exposure to the hazard of concern (e.g., measurements indicating that human exposure occurred and the extent of exposure) and information to identify and link the population of interest with the exposure(s) of concern (e.g., through the use of job titles, deployment dates, and locations or other relevant military characteristics).

Nations across the world bear a collective duty to minimize injury and illness in uniformed members, and to properly compensate and support those whose health is impacted as a result of their service. Government administrations therefore have a vested interest in research that applies strong and current exposure methodologies, to inform prevention and supportive health measures in both active defense and veteran populations.

To support this need, the following sections summarize some key considerations when conducting exposure assessment for research into long-term health impacts in military populations. This includes the identification of hazards related to military service and potentially exposed groups, the collection of data to describe exposures, and the assignment of exposures to estimate health risks. Opportunities and future directions for exposure assessment in this field are also discussed.

## Identifying Hazards and Exposed Groups Across Person, Place and Time

Military technologies, conflicts, and other factors may impact exposures incurred across person, place, and time. This variability can be viewed as a rich opportunity for health research, since epidemiology relies on heterogeneity in exposures across groups in order to assess risk. It can also present unique challenges in differentiating exposure groups, since exposure contexts range from very broad (e.g., deployments that may affect entire regiments, battalions, or ship crews) to specific (e.g., occupational groups or other subsets within the larger population) (14).

Increasing diversity in military populations also confers a need for exposure information to support the health and well-being of various minority groups. For example, females typically represent <20% of NATO Forces personnel (15), however the proportions of females in service and their roles in combat-related activities are expanding (15, 16). While sex and gender influence the military experience, including type and extent of exposure to health hazards (17), little research to date has examined physical exposures in female service members.

The effects of deployment related exposures on military and veteran health is a topic of great interest to the media, public, and decision makers with a prerogative to address the health needs of those who have served on behalf of their countries. Deployment in general (yes/no) is often used as a proxy for exposures associated with deployment, particularly in situations where individual-level information on personnel locations and exposures is missing (18). This approach can be useful as a starting point, since some hazardous exposures may indeed be specific to a particular deployment. For example, Glass and colleagues observed six exposures or indicators of exposures that were significantly more likely to be reported by Australian veterans in relation to the 1991 Gulf War as compared to other deployments (19).

A lack of specific exposure assessment can however be problematic in deployment-focused epidemiological studies. The use of “deployment” as a rough proxy does not define subgroups of personnel who may have incurred more significant exposures during their deployment (e.g., burn pit smoke or blast exposures) (2, 3). It is also inadequate to identify similar or different exposures likely to be incurred outside of deployment. To illustrate, Glass et al. also observed that some exposures listed in Gulf War questionnaires (e.g., engine exhaust, petroleum products, pesticides) are commonly encountered during training, military exercises, and other non-war circumstances (19). Further, the same study noted that proportions of military members deployed by element (e.g., navy, army, air force) may differ across countries for a given conflict, with potential impacts on exposures (for example, respondents who served in the army reported more exposures than their counterparts in the navy or air force) (19).

This underscores the need to consider selection issues from an exposure angle in addition to the potential for “healthy soldier” and “healthy warrior” effects. Where and to what extent hazardous exposures occur may vary across a number of possible scenarios, including:

- Exposures relating to a specific deployment, that are distinct from other deployments or military contexts, e.g., herbicide defoliants used specifically in the Vietnam conflict (20).
- Similar exposures that occur across different deployments [e.g., combat exposure (21, 22)].
- Exposures that are not specific to the military and may occur in various occupational settings and contexts [e.g., solvent use in aircraft maintenance (23)].

When a deployed population is the focus of study, the potential for measurement error may be reduced through incorporation of additional exposure details concerning occupations, duties, and proximity to certain environmental hazards (2, 3). In some instances, where exposure to a hazard is anticipated across deployments or other circumstances (such as military training or exercises), it may be reasonable to pool populations and benefit from larger study numbers. Such strategies may support greater precision and possibly subgroup analyses, such as assessment of health risks in female members or other minority groups.

Veterans in receipt of government compensation or other services, or who belong to veterans' organizations, are likely not representative of the full veteran population. When identifying veteran populations for study, care should be taken to consider the recruitment source and how this may impact on the presence, and reporting, of exposures incurred during military service.

## Exposure Data Sources

Information on work environments, occupations, tasks, and hazards encountered can be used to assess or infer exposures, and to identify surveillance priorities. Primary sources of exposure information include occupational measurements (often referred to as “environmental monitoring data” and sometimes including “biomonitoring data”), surveys or questionnaires, and administrative databases (e.g., military personnel and pay administration systems, medical records, veteran, and pension information systems).

### Exposure Measurements

Exposure measurements can be used to inform various types of research, particularly quantitative analyses focused on exposure-response relationships. Exposure measurements are also useful to inform targeted measures at both the population and individual level (e.g., administrative and engineering controls, personal protective equipment) to reduce harmful exposures in active members (24).

Exposure assessment occurs during occupational and environmental health “intelligence preparation of the battlefield” to provide base-line data prior to force deployment to an operations theater. In theater, routine surveillance continues in line with civilian practice, surveys may be carried out in response to incidents, and post activity reports may give preliminary insights into risks (25). Environmental surveillance procedures required at military camps or bases [e.g., air, water, soil, and bulk sample testing for hazard, or risk management activities (24, 26, 27)], may serve a different purpose than the collection of measurements to serve as individual-level exposure proxies in epidemiological studies. For example, some defense risk assessment models place greater emphasis on acute effects that may impact operations in the short term, rather than on delayed health effects (25).

Environmental monitoring (e.g., measurement of a substance in air or water, or on body surfaces) may be conducted at the individual level, such as personal air or skin wipe sampling, or through “area” measurements, such as stationary air sampling proximal to personnel. Area measurements can be useful as a screening tool to identify health threats to deployed military personnel, but serve less well as individual-level exposure proxies in epidemiological studies (3). Personal-level exposure measurements are highly relevant to inform analytic models to predict exposure risk or disease in time and space, and to validate the development of other tools, such as job exposure matrices (28, 29). Such data may also be linked to individual medical records, or used to cross reference job and operation specific exposure matrices.

Though more invasive than environmental monitoring, biomonitoring (i.e., measuring biological indicators of exposure in human blood, urine, or other media) can be used to identify internal exposure to a hazard, and to evaluate the efficiency of protective measures (30). Biomarkers are not available for all hazards, however. They are also less informative to pinpoint the source of exposure (3), such as when a hazard occurs both at home and work, or across multiple media (e.g., in both air and water). Environmental monitoring permits the quantitative evaluation of chronic health risks, which requires unit risk values, and is also preferred for assessing chemical hazards with short half-lives (30).

It is well-established that occupational exposures vary both between and within individuals (31). Between individuals with the same job title, the combination of tasks and time spent on them can vary significantly, while exposure variability within individuals is expected due to changes in tasks and environmental conditions over time (31, 32). Exposure measurement strategies based on individual vs. grouped measurement strategies each have benefits and disadvantages. Individual-based measurement strategies generally increase precision of exposure-response relationships, at the expense of introducing bias and effect attenuation (33, 34). Group-based exposure assessment is commonly used in occupational epidemiological studies since data on individual exposures may be missing (i.e., when assessing retrospectively) or can be impractical/costly to collect (35). Grouped exposure assessment can provide reasonably unbiased estimates of exposure-response relationships, since the expected error (overestimation or underestimation of some group members' exposures) results in less exposure-response attenuation as compared to each individual being assigned the mean of their own exposure measurements (a Berkson error structure) (33, 34). The validity of a grouped measurement approach to exposure assessment approach relies on the assumption that individuals within assigned groups are similarly exposed (35), and that assigned groups provide sufficient exposure contrast (36).

### Questionnaires/Surveys

Researchers may also use self-reported data to assess associations between exposures and health outcomes in military and post-military populations. Information on exposure can be collected from serving members or veterans through various forms of questionnaires and surveys, both prospectively (e.g., through pre/post deployment questionnaires) and retrospectively (e.g., post-military surveys). This approach to collecting exposure information can be particularly useful to obtain information on occupational histories and exposure circumstances that would be otherwise unavailable. Exposure scales based on self-reported data have been developed to assess various types of hazards in military studies, including chemical and environmental exposures (19, 37) and psychosocial risk and resilience factors related to various operational contexts (22, 38, 39). The format of survey administration (e.g., web-based vs. in-person or telephone interviews) has been shown to impact on the reliability of exposure data collected and generalizability of results (40, 41).

Reporting bias (when exposure is attributed more frequently by those who are ill vs. those who are not), can be an issue in self-reported exposure assessment. As outlined elsewhere (1), bias in exposure reporting has been described by several Gulf War investigators, and may occur for various reasons (e.g., in relation to one's health status or as a result of bias in perception, recall, or other factors). As one example, a US study of 227 Gulf War soldiers found that only 24% of those told about their potential exposure to hexavalent chromium reported the event accurately in their post-deployment forms, while only 42% mentioned chemical exposure of any kind (37). Reliability of self-reported exposures over time, as assessed through test-retest measurements, may be stronger for some hazards and poorer for others (42). It has been noted that the reporting of military hazards after a conflict can be unstable, and may relate to one's current self-rated perception of health (43). Individuals seeking benefits and/or compensation may report exposures differently than those who are not; the potential for bias here may be reduced through confidential assessments where participants are made aware that their responses will not affect benefits eligibility.

Data quality and comparability across data sets may also be increased through the use of assessment tools with demonstrated reliability and validity (44). Further, certain types of exposure information collected through questionnaires and surveys has been found to be accurate and valid. Self-reported data for some work schedules and occupations have been found to correlate well with objective sources in both civilian and military research, respectively (45, 46). The validity of self-reported occupational histories is generally high, which for some hazards can be used to assign exposures with a reasonable degree of accuracy (44). The use of military occupation as “exposure” does not identify specific agents as risk factors, and may mask the effect of an agent to which only some individuals in the job are exposed. However, where there is sufficient exposure contrast, the use of job title or other broad category can be useful in situations where exposures to complex mixtures or environments with multiple hazards are of interest, as often occurs in the military context.

### Administrative Data

Various types of administrative datasets may be used to define exposure subgroups or build occupational histories for use in reconstructing potential exposures. In Canada for example, the Department of National Defence's pay system records may be merged with other human resources data to identify particular cohorts of interest and build a military work history for personnel (47). Australian Defense Force vaccination records have been used in validation research focused on health outcome analyses (48). Nominal roll data (lists of armed forces members who served in a particular capacity) have also been used to identify exposure to service in certain regions or conflicts (42).

The use of administrative data to assist exposure assessment must be accompanied by data quality initiatives to ensure that the data are accurate and valid. The use of nominal rolls to identify deployed members may for example be limited by issues of accuracy, as demonstrated by the finding that 8.5% of veterans from a US Gulf War nominal roll sample did not recall being deployed (42). Some forms of pay data provide the strength of a built-in feedback mechanism whereby both personnel and the employer are motivated to correct pay errors as soon as possible (47). However, differential retention of personnel data by rank and service has also been noted, with the potential to bias retrospective studies of exposure-effect relationships since exposures may differ across these factors (49).

## Combining Data Sources to Assign Exposures

Regardless of the data collection strategy, some degree of expert opinion is required to assign exposures in an epidemiological study. For instance, measurement data indicating the general extent of exposure expected with a certain job or task may be combined with information on type of job or tasks performed in order to quantitatively or qualitatively estimate an individual's exposures over space and time.

The selection of an appropriate exposure indicator for use in epidemiological studies has implications for observed associations between exposure and disease, however, this decision is often not straightforward (28, 50). Since the mechanisms linking exposures to health outcomes are often unclear, the use of multiple metrics can be useful (50). Qualitative and semi-quantitative (e.g., low, medium, high) measures or quantitative metrics (e.g., exposure duration or cumulative exposure) may be applied. Cumulative exposure measures are commonly used in chronic disease studies, whereas short duration (peak) exposure measures may be most appropriate to assess acute effects. The application of quantitative or semi-quantitative exposure data in long-term occupational studies is a widely advocated strategy that allows for exposure-response analyses and identification of risks even at low levels of exposure (51, 52).

Job exposure matrices (JEMs) are a long standing and widely used tool to estimate exposures in occupational health studies. Job exposure matrices essentially allow for exposure to be estimated using job histories, which can be specific to a military element or other broad characteristic. A typical JEM consists of a job axis (e.g., occupational codes) and an exposure axis (e.g., probability or extent of exposure = low/medium/high). Recently some quantitative JEMs have also been developed through calibration with measurement data (53). Once developed, JEMs are economical and relatively straightforward to apply, with any exposure misclassification expected to be non-differential with respect to the health outcome of interest (33). Thus, JEMs offer the advantage of using job histories to estimate exposures in a systematic and unbiased way, with an efficient and reproducible methodology (54). Given the wide variety of occupations and exposure circumstances present in the military context, the use of JEMs as an exposure assessment tool may be useful, although they must be developed with recognition of their limitations (54). In civilian contexts, various types of task-based JEMs have been developed when job title alone was insufficient to describe particular work or environmental circumstances that influenced exposures (54). For military studies, information on the element (e.g., navy, army, or air force), component (e.g., regular or reserve forces), or deployment factors could be applied to increase a JEM's informativeness, through specific modules. This supports the need to collect, compile, and retain information on military members' occupational histories.

## Moving Forward

Recent technological advances are facilitating the development of new exposure assessment methods that build upon traditional approaches. These include geospatial information systems (GIS), portable and personal sensing (e.g., smartphone-based sensors and assessments), and Internet-based platforms to support self-reported questionnaire assessments (55).

Genomics has been proposed as an important discipline in the future of exposure assessment. The “exposome” concept, first proposed in 2005 (56), refers to every exposure (both internal and external) encountered by an individual from conception to death. Though in its early stages, it is hoped that this concept will provide more complete environmental exposure assessment in epidemiological studies, alongside targeted exposure assessment approaches focused on individual agents (57) “Top down” and “bottom up” approaches have been suggested; the former to detect epigenetic changes in tissues, then search for exposures, the latter to focus on exposures and assess internal effects. Given that such analyses require “big data,” the military setting, with various exposures and potentially large numbers of individuals exposed, may be an appropriate milieu to examine applications of the exposome concept in epidemiological studies (58).

In order to optimize the effectiveness and quality of new and ongoing military and veteran health research and surveillance systems, multidisciplinary collaboration is needed between government departments, researchers in and outside of government, and medical professionals (24, 59) Defense administrations may be most keenly aware of emerging military exposures (e.g., new personal protective technologies) and are likely to have the most direct access to study populations for current or prospective assessment. Follow up of personnel after their release from service, often the domain of veterans' administrations, is also a vital piece of successful research given the long latencies between various exposures and chronic health outcomes. Limited data accessibility across departments can be a challenge, particularly when attempting to link exposure data held by Defense with health outcomes data held by Veterans' Affairs. The potential benefits of informing both prevention and compensation policies points to the need for systems that facilitate data access and exchange between defense and veterans' administrations (59, 60). Routine debriefings and information exchange on potential hazards and exposures, particularly following deployments, could also be used to strengthen research connections across departments. Expertise from occupational health researchers and other experts outside of government should be leveraged to maximize research impact.

The storage of exposure information in centralized database formats is also important to facilitate use and accessibility over time. A number of countries are working to develop methodologies for linking military service records with records from veterans administrations, survey data, and other types of information (18, 61) Such linkages provide new opportunities to follow individuals over time and reduce loss to follow up, which is a common and major limitation in long-term health studies. Leveraging data sources through linkages (e.g., combining data on service characteristics with cancer registries, hospital records, or population-level surveys) also increases the feasibility of studying less understood subgroups within military and veteran populations. The ability to disaggregate by element, sex, or other characteristics of interest will permit the identification of subgroups experiencing higher or lower risk of disease, as well as factors to target through prevention measures (59).

While the current paper has focused primarily on physical exposures in relation to health, it should be noted that many of the same concepts are relevant in research examining links between psychological exposures and health, a topic area that has received increasing research interest in recent years (62–64).

## Summary

Exposure assessment is an integral aspect of population-level health research. It is particularly challenging in epidemiological research focused on the long term impacts of military service, where exposure pathways are not well-defined, latency periods are long, and many connections between military hazards and health are not well-established.

There is no “correct” approach to conducting exposure assessment in the military and veteran context. As with any occupational study, the choice of exposure groups, data sources, and methods to assign exposures requires careful consideration about the hazard of interest and hypotheses regarding its relationship to the health effect(s) under study.

Moving ahead, a stronger understanding of links between military exposures and long-term health will be supported by new ideas and technologies as well as collaboration across research disciplines, health professionals, and government departments. The strength of analyses will also rely on the ability to collect and centralize data that describe exposures incurred both during military service and throughout the life course, for linkage with health information. Such initiatives, which rely heavily on support from upper levels of defense and veterans' administrations, are essential to develop new research knowledge to protect the health of current and former military personnel.

## Author Contributions

AH conceptualized the manuscript and wrote the first draft. MM, LV, DM, and DG contributed to manuscript content and revisions. All authors approved the submitted version of the article.

## Conflict of Interest

The authors declare that the research was conducted in the absence of any commercial or financial relationships that could be construed as a potential conflict of interest.
